# Supplementary material for: Antibacterial Effect of Carbon Nanomaterials: Nanotubes, Carbon Nanofibers, Nanodiamonds, and Onion-like Carbon
Source: Materials (Basel). 2023 Jan 19;16(3):957. doi: 10.3390/ma16030957 (PMC9918274; doi:10.3390/ma16030957)
Supplement: Supplementary file 1 [file materials-16-00957-s001.zip › materials-2125609-supplementary.pdf]

The appendix contains TEM images of NCMs used in experiments.

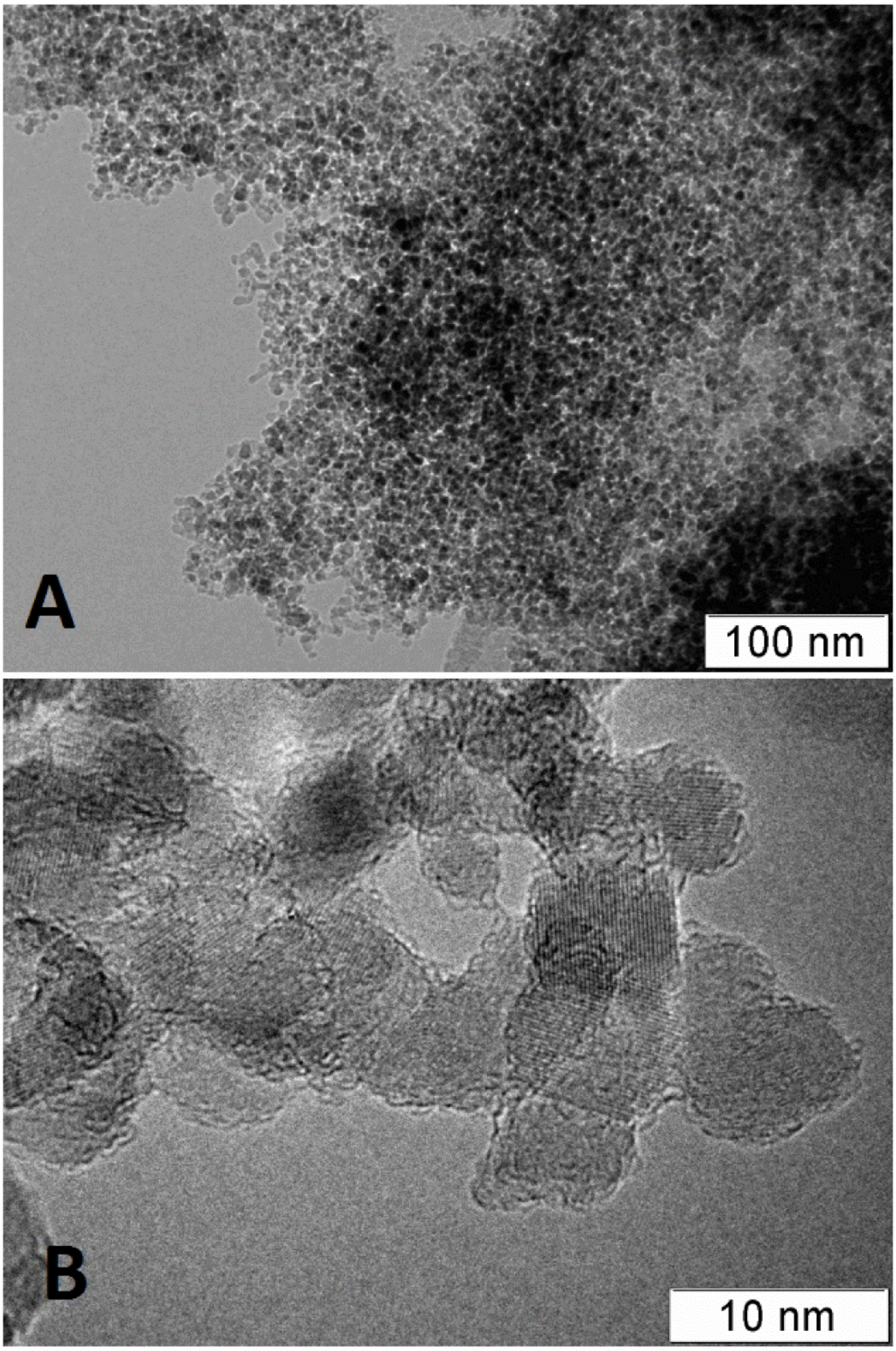

Figure S1. TEM micrographs of NDs (“Altay”, Russia)

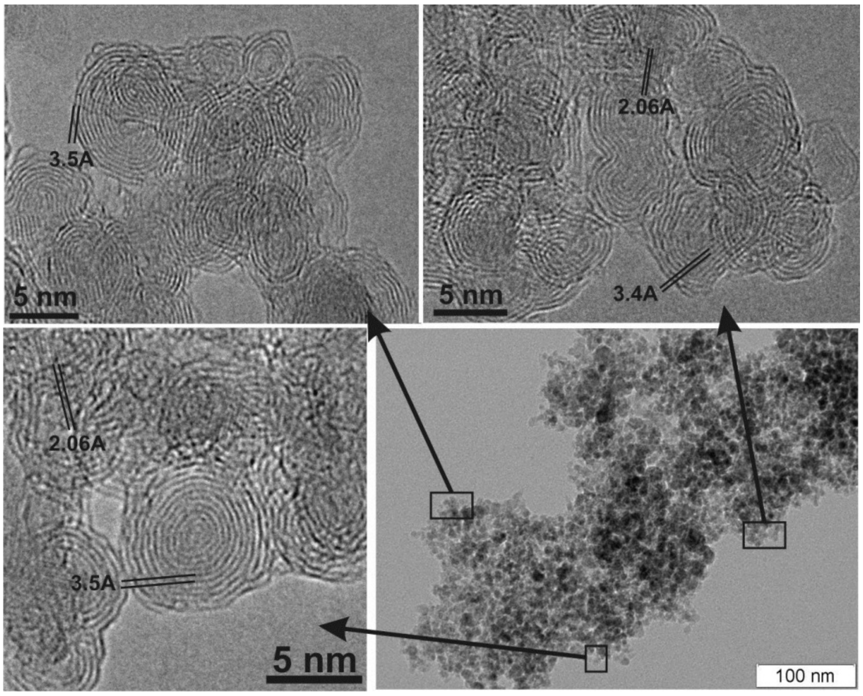

Figure S2. TEM micrographs of OLC prepared by the ND thermal treatment at 1527 °C under vacuum.

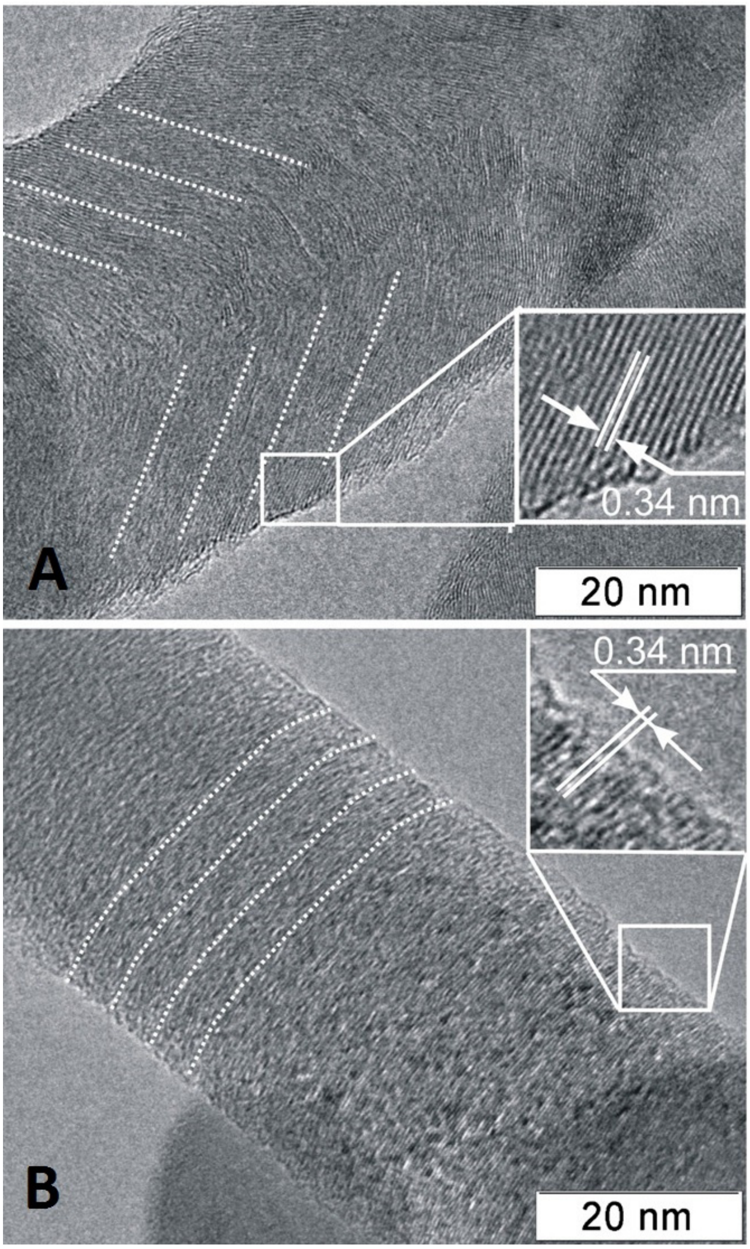

Figure S3. TEM micrographs of CFC-1 (A) and CFC-2 (B)

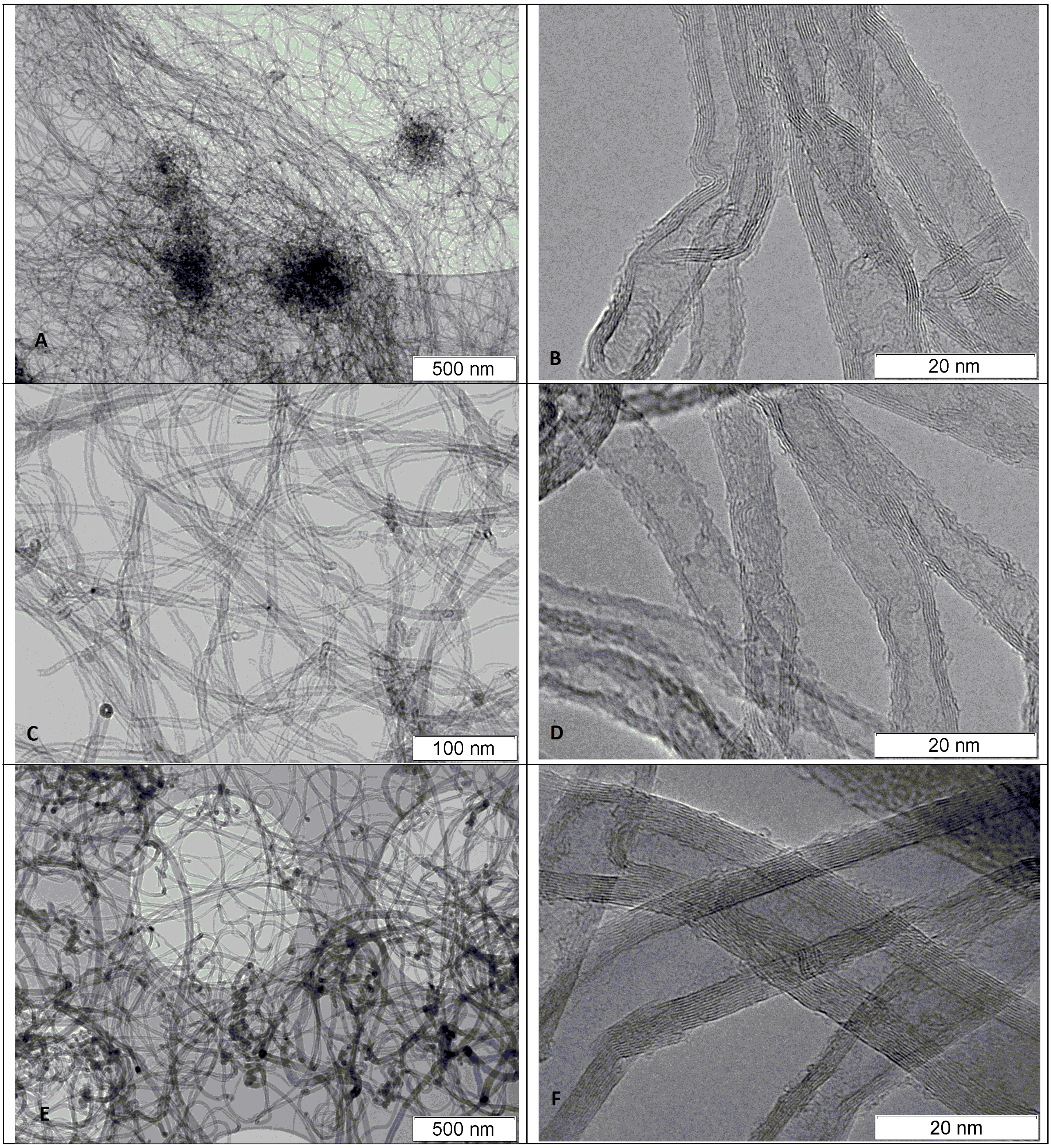

Figure S4. TEM micrographs of MWCNT-1 (A, B), MWCNT-2 (C,D) and MWCNT-3 (E,F)
